# Agreement Between WHO and Thai Fetal Growth Charts for Classifying Estimated Fetal Weight in Thai Singleton Pregnancies: A Retrospective Cross‐Sectional Study

**DOI:** 10.1155/ogi/6968622

**Published:** 2026-09-07

**Authors:** Fuanglada Tongprasert, Jitada Tanasuwankasem, Pimtawan Jatupornpakdee, Primrose Tothanarungroj, Supapitch Sirimangklanurak, Theera Tongsong

**Affiliations:** ^1^ Department of Obstetrics and Gynecology, Faculty of Medicine, Chiang Mai University, Chiang Mai 50200, Thailand, cmu.ac.th; ^2^ Fetal Center, Faculty of Medicine, Chiang Mai University, Chiang Mai 50200, Thailand, cmu.ac.th; ^3^ Faculty of Medicine, Chiang Mai University, Chiang Mai 50200, Thailand, cmu.ac.th

**Keywords:** estimated fetal weight, fetal growth chart, large for gestational age, small for gestational age, WHO standards

## Abstract

**Background and Aims:**

Universal fetal growth standards may classify fetal size differently from locally derived charts, particularly in populations with different anthropometric characteristics. This study aimed to compare the agreement and classification patterns of the WHO and Thai fetal growth charts when applied to the same ultrasound‐derived estimated fetal weight (EFW) values in a Thai clinical cohort.

**Methods:**

This retrospective analytical study included 1109 Thai pregnant women between 21 and 42 weeks of gestation who received antenatal care at the Faculty of Medicine, Chiang Mai University. Ultrasound biometric parameters—biparietal diameter, head circumference, abdominal circumference, and femur length—were used to estimate fetal weight via the Hadlock‐3 formula. EFW percentiles were calculated using both the WHO fetal growth standards and the Thai fetal growth chart. The proportions of fetuses classified as SGA (< 10th percentile) and LGA (> 90th percentile) were compared.

**Results:**

The WHO chart classified a significantly higher proportion of fetuses as SGA (27.1%) compared to the Thai chart (12.7%) (*p* < 0.001), while identifying fewer fetuses as LGA (3.3% vs. 5.4%, *p* < 0.001). Although the percentile rankings from the two charts were significantly correlated (Spearman’s correlation coefficient of 0.750, *p* < 0.001), the correlation was moderate. Bland–Altman analysis indicated relatively poor agreement between the two methods, with discrepancies that may be clinically relevant.

**Conclusion:**

The WHO and Thai fetal growth charts produced different fetal size classifications in this cohort, with the WHO chart classifying more SGA and fewer LGA fetuses, particularly in late gestation. Further outcome‐based validation is needed to determine the clinical performance of each chart.

## 1. Introduction

Fetuses classified as small for gestational age (SGA) are at increased risk for adverse perinatal outcomes, as this condition often reflects fetal growth restriction (FGR), in which the fetus fails to achieve its full growth potential [1]. SGA is typically diagnosed when the estimated fetal weight (EFW), derived from prenatal ultrasound, falls below the 10th percentile for gestational age (GA) [1]. Conversely, large for gestational age (LGA) fetuses, defined by an EFW above the 90th percentile, are associated with elevated risks of shoulder dystocia, birth trauma, and cesarean delivery [2]. Accurate classification is essential, as both underdiagnosis and overdiagnosis may lead to suboptimal care—either through missed interventions or unnecessary procedures, added healthcare costs, and maternal anxiety.

EFW is estimated using ultrasound‐based formulas incorporating biometric parameters such as biparietal diameter (BPD), head circumference (HC), abdominal circumference (AC), and femur length (FL). The Department of Obstetrics and Gynecology, Faculty of Medicine, Chiang Mai University, currently uses the Hadlock third formula (Hadlock‐3) [3], one of the most widely accepted methods for fetal weight estimation, routinely embedded in standard ultrasound equipment.

EFW percentiles are then interpreted using fetal growth charts, such as those developed by Hadlock [4], the World Health Organization (WHO) [5], INTERGROWTH‐21st (IG‐21) [6], and the Eunice Kennedy Shriver National Institute of Child Health and Human Development (NICHD) [7]. Although there is no global consensus on the optimal chart, the WHO standard—derived from a multiethnic cohort across 10 countries, including Thailand—is widely used in clinical practice and research. Despite this, our institution has used a locally developed Thai fetal growth chart since 1993 [8]. This chart was developed from local data at Maharaj Nakorn Chiang Mai Hospital and is not an individually customized chart adjusted for maternal or fetal characteristics. However, the accuracy and clinical utility of the Thai chart in classifying SGA and LGA fetuses have not been validated. Although several local fetal growth charts have been published [9–13], evidence regarding their agreement with global standards, such as the WHO fetal growth chart, remains limited. This study aimed to compare the WHO and Thai fetal growth charts in determining EFW percentiles and classifying fetal size when both charts were applied to the same Thai clinical cohort.

## 2. Materials and Methods

### 2.1. Study Design and Subjects

This study was conducted as an analytical, retrospective cross‐sectional study. The study population consisted of pregnant women who received antenatal care at Maharaj Nakorn Chiang Mai Hospital, a tertiary university hospital, between July 1, 2022, and December 31, 2023. Ethical approval was obtained from the institutional review board (Research ID: OBG‐2567–0171). Informed consent was not obtained because this was a retrospective analysis of anonymized data, and the requirement for consent was waived by the institutional ethics committee. Inclusion criteria were as follows: Thai ethnicity, singleton pregnancy, first‐trimester dating confirmed by crown–rump length measurement, and GA between 21 and 42 weeks at the time of ultrasound examination. Exclusion criteria included fetal death, fetal anomalies, chromosomal abnormalities, incomplete fetal biometric data, and abnormal findings related to the amniotic fluid, placenta, or umbilical cord. The study cohort included both low‐ and high‐risk singleton pregnancies encountered in routine clinical practice. All pregnancies meeting the predefined eligibility criteria during the study period were included, irrespective of their baseline obstetric risk status. As this was a retrospective study, all eligible cases during the study period were included. To assess sample size adequacy, we assumed expected proportions of 10% for both SGA and LGA, based on their conventional definitions as EFW below the 10th percentile and above the 90th percentile, respectively. Using a 95% confidence level and an absolute precision of 2.0%, the minimum required sample size was calculated to be 865 pregnancies.

### 2.2. Data Curation

Fetal and maternal data were retrieved from the hospital’s ultrasound digital database and electronic medical records. In cases where women underwent multiple ultrasound examinations during pregnancy, only the final examination performed within 2 weeks before delivery was included in the analysis. Fetal ultrasound parameters included GA (in weeks and days), BPD, HC, AC, and FL, all measured in millimeters. These biometric parameters were used to calculate ultrasound‐derived EFW. Because a single ultrasound‐derived EFW was used for each pregnancy, serial fetal growth velocity was not evaluated. All ultrasound examinations were performed by maternal–fetal medicine (MFM) fellows under supervision or by attending physicians, following institutional imaging protocols, using GE Voluson E8, Voluson E10, or Voluson Expert 22 scanners (GE Medical Systems, Zipf, Austria), equipped with abdominal transducers with frequency ranges of 2–5 MHz or 2–9 MHz, depending on the ultrasound system and probe used. To ensure consistency and minimize interobserver variability, all sonographers underwent structured training, and all examinations were performed under quality‐controlled conditions, including regular image audits on a sampling basis. EFW was calculated using the Hadlock‐3 formula, automatically generated by the ultrasound machines based on biometric parameters: [Log_10_ weight = 1.326 – 0.00326(AC x FL) + 0.0107(HC) + 0.0438(AC) + 0.158(FL)]. EFW percentiles were then determined using both the WHO fetal growth chart, via the online WHO Fetal Growth Calculator (https://srhr.org/fetalgrowthcalculator/#/), and the Thai fetal growth chart derived from a Thai population model [8], as presented in Supporting Table S1. The Thai chart calculates the predicted mean and standard deviation (SD) based on GA using the following equations:

• *Predicted mean = −1723.668774,703,609 + 59.37904432,524,342(GA) + 1.531500564,652,684(* *G* *A* ^2^ *)*
• *Predicted SD = 16.72198051,948,052(GA) −327.8038636,363,638*
• *Percentile = 100/(1 + e^(-1.7* × *Z-score))*

### 2.3. Data Analysis

Statistical analyses were performed using the Statistical Package for the Social Sciences (SPSS), Version 30.0 (IBM Corp., Released 2024; IBM SPSS Statistics for Windows, Version 30.0, IBM Corp., Armonk, NY, USA). Agreement between the percentile values derived from the WHO and Thai fetal growth charts was assessed using Spearman’s correlation coefficient and Bland–Altman plots. The proportions of fetuses classified as SGA and LGA by each method were compared using McNemar’s chi‐square test. The distribution of continuous variables was evaluated using the one‐sample Kolmogorov–Smirnov test. All statistical tests were two‐sided, and a *p* value of < 0.05 was considered statistically significant.

### 2.4. Use of Artificial Intelligence Tools

ChatGPT (OpenAI, GPT‐5.5 Thinking) was used to assist with language editing, improving clarity, and revising selected portions of the manuscript and responses to reviewers. The tool was not used for study design, data collection, data analysis, interpretation of results, or generation of figures or tables. All AI‐assisted content was critically reviewed, edited, and verified by the authors, who take full responsibility for the final content of the manuscript.

## 3. Results

A total of 6674 cases were initially reviewed. After applying the inclusion and exclusion criteria, a total of 3529 cases were excluded, primarily due to the following reasons: incomplete biometric data (57.7%), multiple gestations (36.4%), fetal structural or chromosomal abnormalities, and abnormal placental, amniotic fluid, or umbilical cord findings (5.9%). An additional 2036 cases were excluded due to repeated measurements and measurements taken before 21 weeks of gestation, which were outside the applicable range of our growth chart. Consequently, 1109 cases met the eligibility criteria and were included in the final analysis. Of these, 307 cases (27.7%) were in the second trimester, and 802 cases (72.3%) were in the third trimester. Due to non‐normal data distribution, descriptive statistics are presented as medians and interquartile ranges (IQRs). The median GA at ultrasound examination was 32 weeks (range: 21–42 weeks; IQR: 11.0 weeks). The median EFW at ultrasound examination was 1689 g (range: 192–4420 g; IQR: 1659 g).

**FIGURE 1:**
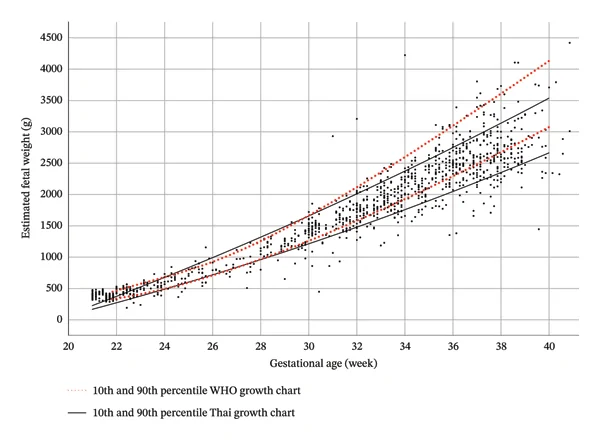
Scatterplots of estimated fetal weight (EFW) by gestational age with 10th and 90th percentile curves from the WHO and Thai fetal growth charts.

The WHO fetal growth chart identified a significantly higher proportion of fetuses as SGA compared to the Thai fetal growth chart (27.1% vs. 12.7%; *p* < 0.001), as shown in Table 1. In contrast, the WHO chart identified fewer fetuses as LGA than the Thai chart (3.3% vs. 5.4%; *p* < 0.001). Subgroup analysis by GA revealed that the WHO chart consistently identified a higher prevalence of SGA than the Thai chart across all gestational windows: late second trimester (21–27 weeks), early preterm (28–33 weeks), late preterm (34–36 weeks), and term (37–41 weeks), as presented in Table 2 and Figure 1. Notably, the discrepancy in SGA classification between the two charts widened with advancing GA. Similarly, the WHO chart identified a significantly lower prevalence of LGA than the Thai chart in all gestational subgroups.

**TABLE 1:** Comparison of fetal growth classification using the WHO and Thai fetal growth charts.

Classification	WHO growth chart (*N* = 1109)		Thai growth chart (*N* = 1109)		*p* value^∗^
	*N*	%	*N*	%	
SGA	300	27.1	141	12.7	< 0.001
AGA	772	69.6	908	81.9	—
LGA	37	3.3	60	5.4	< 0.001

Abbreviations: AGA, average for gestational age; LGA, large for gestational age; SGA, small for gestational age.

^∗^The proportions were compared using McNemar’s chi‐square test.

**TABLE 2:** Fetal growth classification by gestational age group using the WHO and Thai fetal growth charts.

GA group	Diagnosis	WHO growth chart (*N* = 1109)		Thai growth chart (*N* = 1109)		*p* value^∗^
		*N*	%	*N*	%	
21–27 weeks (*N* = 308)	SGA	73	23.8	50	16.3	< 0.001
	AGA	222	72.3	235	76.5	—
	LGA	12	3.9	22	7.2	0.008

28–33 weeks (*N* = 348)	SGA	71	20.4	17	4.9	< 0.001
	AGA	271	77.9	322	92.5	—
	LGA	6	1.7	9	2.6	0.375

34–36 weeks (*N* = 252)	SGA	51	20.2	27	10.7	< 0.001
	AGA	193	76.6	213	86.1	—
	LGA	8	3.2	12	4.8	0.125

37–41 weeks (*N* = 202)	SGA	105	52.0	47	23.3	< 0.001
	AGA	86	42.6	138	68.3	—
	LGA	11	5.4	17	8.4	0.063

Abbreviations: AGA, average for gestational age; GA, gestational age; LGA, large for gestational age; SGA, small for gestational age.

^∗^The proportions were compared using McNemar’s chi‐square test.

**FIGURE 2:**
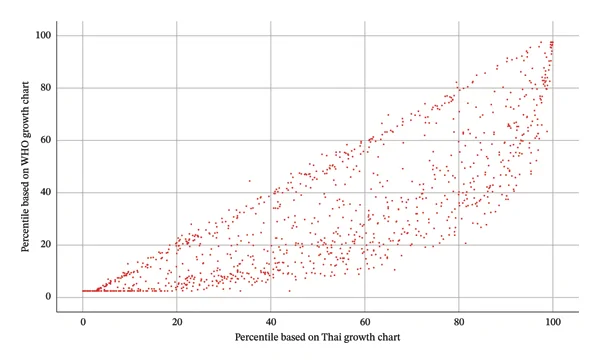
Scatter plot showing the correlation between estimated fetal weight (EFW) percentiles derived from the WHO and Thai fetal growth charts. The Spearman correlation coefficient (*ρ* = 0.750; *p* < 0.001).

**FIGURE 3:**
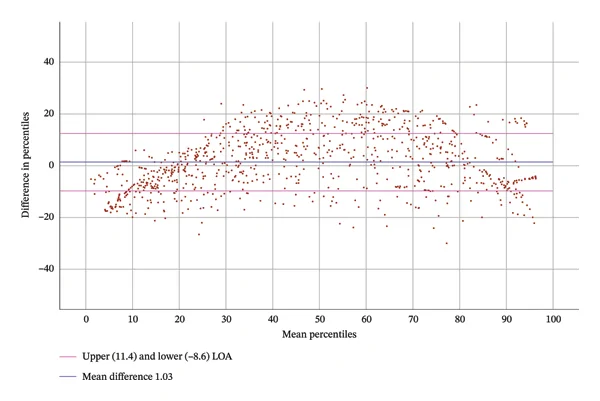
Bland–Altman plot comparing EFW percentiles between the WHO and Thai fetal growth charts.

The percentile rankings derived from the two fetal growth charts were significantly correlated, with a Spearman’s correlation coefficient of *ρ* = 0.750 (*p* < 0.001), indicating a moderate positive association (Figure 2). However, Bland–Altman analysis revealed relatively poor agreement between the two methods, with a mean difference of 1.03 percentile points and 95% limits of agreement ranging from −8.6 to +11.4. Notably, more than 10% of data points fell outside these limits (Figure 3). Discrepancies in percentile rankings were particularly evident in late gestation.

## 4. Discussion

This study yielded several important findings: (1) The WHO fetal growth chart classified a significantly higher proportion of fetuses as SGA and a lower proportion as LGA compared to the locally derived Thai growth chart. (2) Although there was a moderate correlation between percentile values generated by the two charts, notable discrepancies were observed—especially in late gestation. (3) The classification of fetal size differed substantially between the two charts, suggesting that chart selection can materially influence fetal size classification in this clinical cohort. Importantly, this study was not designed to determine whether one growth chart is superior to the other. Rather, it demonstrates that the choice of fetal growth chart can substantially influence prenatal fetal size classification. In our population, the WHO fetal growth chart classified a greater proportion of fetuses as SGA, whereas the Thai chart produced SGA and LGA proportions closer to the expected 10% percentile‐based cutoffs. These findings should therefore be interpreted as differences in classification patterns between the two charts, rather than evidence of diagnostic superiority or clinical performance.

These differences likely stem from the populations used to develop the charts. The Thai chart was based on a local population [8], whereas the WHO chart was constructed from a multiethnic cohort that included only a small subset of Thai participants (124 out of 1439 women) [5]. The limited agreement observed in this study indicates that the two charts produced different classification patterns in this cohort. However, because neonatal outcomes were not assessed, our findings cannot determine which chart provides better clinical prediction or diagnostic accuracy. Nevertheless, our findings align with recommendations from the FIGO Safe Motherhood and Newborn Health Committee, which advocate for the use of local or regional growth charts where available [14].

In an unselected population, the prevalence of SGA and LGA would be expected to approximate 10% only when the growth chart appropriately represents the target population, consistent with their definitions as below the 10th and above the 90th percentile, respectively [15]. In this study, however, the observed prevalence of SGA exceeded this threshold for both growth charts—particularly for the WHO chart—while the prevalence of LGA was lower than expected. This discrepancy may reflect selection bias, as fetuses with suspected growth abnormalities are more likely to undergo ultrasound in routine clinical care [16]. Although a universal midtrimester screening policy was in place and efforts were made to limit bias by including only one scan per case, some degree of selection bias remains likely. Therefore, these findings should be interpreted with caution, consistent with prior research recommending outcome‐based validation to ensure accurate classification of fetal growth abnormalities [17].

The study’s strengths include a paired design and a sufficiently sized dataset for comparative analysis, with both charts applied to the same cases using identical fetal weight estimates derived from the Hadlock‐3 formula. This minimized variability and enhanced internal validity. In addition, the study focused on prenatal fetal size classification rather than postnatal birthweight alone, reflecting the clinical context in which fetal growth charts are commonly applied before delivery. However, several limitations should be acknowledged. The retrospective design and unequal distribution of GAs—particularly fewer cases at later gestation—may limit generalizability. The WHO chart was originally developed using prospective, longitudinal data, while the Thai chart was derived from a cross‐sectional study—differences that may affect percentile comparisons. However, both charts were retrospectively applied to the same dataset in this study; thus, potential bias related to the original chart development methods is unlikely to have influenced our findings. Neonatal birthweight, neonatal morbidity, and postnatal classification as SGA or LGA were not available in the analytic dataset. Therefore, this study could not assess sensitivity or specificity or develop an outcome‐based prediction model for adverse neonatal outcomes. Additionally, selection bias related to referral patterns for ultrasound remains a concern. Notably, the study population included both low‐ and high‐risk groups, reflecting real‐world clinical practice. This is in fact the appropriate target population, as growth charts are theoretically expected to yield comparable results across both risk categories, with their application being particularly crucial in high‐risk pregnancies. Importantly, both charts were applied to the same cohort, regardless of risk status, allowing for a direct and perfect comparison (both charts applied to the same case at the same time). Thus, agreement of results could be assessed within and across both low‐ and high‐risk groups.

Despite efforts to create global fetal growth standards, our findings highlight significant discrepancies between universal and local charts in identifying abnormal growth. These discrepancies may partly reflect differences in the source populations used to construct the WHO and Thai fetal growth charts. A direct week‐by‐week comparison reveals that Thai fetuses [8, 18] are generally smaller than those represented in the WHO chart. Consequently, the WHO chart tends to classify more fetuses as SGA and fewer as LGA due to its relatively lower and higher thresholds, respectively. Our findings are consistent with previous studies demonstrating that fetal growth charts derived from different source populations may produce different fetal size classifications [19–22]. Nevertheless, in the absence of outcome‐based validation, our results should not be interpreted as evidence favoring one chart over the other.

Ultimately, determining which chart is superior requires outcome‐based validation studies, including neonatal birthweight and morbidity data. The development and refinement of population‐specific fetal growth charts should be encouraged. Although FIGO recommends using universal standards with locally adjusted thresholds when no local chart exists [22], this approach risks under‐ or overdetection. In clinical settings, establishing reliable, population‐specific thresholds remains essential but challenging. In light of these findings, future research should consider multicenter collaborations across Southeast Asian countries with similar ethnic backgrounds to establish adequately powered, regionally tailored fetal growth standards that reflect population‐specific characteristics.

## 5. Conclusion

This study demonstrated that the WHO and Thai fetal growth charts yield different classifications of abnormal fetal growth in a Thai population. The WHO chart identified more SGA and fewer LGA fetuses compared to the locally derived Thai chart, with discrepancies increasing in later gestation. These findings indicate that the two charts produce different fetal size classifications. However, because neonatal birthweight and clinical outcomes were not assessed, this study cannot determine which chart is clinically superior. Further outcome‐based diagnostic accuracy studies incorporating clinically important perinatal outcomes are needed to compare the sensitivity, specificity, and predictive values of the two charts.

## Funding

This study was supported by funding from Chiang Mai University.

## Disclosure

Fuanglada Tongprasert affirms that this manuscript is an honest, accurate, and transparent account of the study being reported; that no important aspects of the study have been omitted; and that any discrepancies from the study as planned have been explained.

All authors have read and approved the final version of the manuscript. Fuanglada Tongprasert had full access to all of the data in this study and takes complete responsibility for the integrity of the data and the accuracy of the data analysis.

The funder had no role in study design; data collection, analysis, or interpretation; writing of the report; or the decision to submit the article for publication.

## Conflicts of Interest

The authors declare no conflicts of interest.

## Supporting Information

Additional supporting information can be found online in the Supporting Information section.

## Supporting information


**Supporting Information** Supporting Table S1. Thai fetal growth chart based on Tongsong et al. [8], presenting estimated fetal weight percentiles (10th, 50th, and 90th) by gestational age in weeks, derived from locally developed reference equations.

## Data Availability

The data supporting the findings of this study are not publicly available because they are derived from hospital medical records and are subject to institutional ethics and privacy restrictions. Deidentified data may be available from the corresponding author upon reasonable request and ethics committee approval.
