# Supplementary material for: Agreement Between WHO and Thai Fetal Growth Charts for Classifying Estimated Fetal Weight in Thai Singleton Pregnancies: A Retrospective Cross‐Sectional Study
Source: Obstet Gynecol Int. 2026 Sep 7;2026:6968622. doi: 10.1155/ogi/6968622 (PMC13550868; doi:10.1155/ogi/6968622)
Supplement: Supplementary file 1 [file OGI-2026-6968622-s001.docx]

**Supplement Table 1:** Thai fetal growth chart based on Tongsong et al. (1993): Estimated fetal weight (EFW) percentiles (10th, 50th, and 90th) by gestational age (GA) in weeks, derived from locally developed reference equations

| **GA (week)** | **EFW (grams)** | | |
| --- | --- | --- | --- |
|  | **10th centile** | **50th centile** | **90th centile** |
| 21 | 169 | 199 | 229 |
| 22 | 273 | 324 | 375 |
| 23 | 380 | 452 | 525 |
| 24 | 490 | 584 | 678 |
| 25 | 603 | 718 | 834 |
| 26 | 719 | 856 | 992 |
| 27 | 838 | 996 | 1154 |
| 28 | 960 | 1140 | 1319 |
| 29 | 1085 | 1286 | 1487 |
| 30 | 1214 | 1436 | 1659 |
| 31 | 1345 | 1589 | 1833 |
| 32 | 1479 | 1745 | 2010 |
| 33 | 1617 | 1904 | 2190 |
| 34 | 1758 | 2066 | 2374 |
| 35 | 1901 | 2231 | 2560 |
| 36 | 2048 | 2399 | 2750 |
| 37 | 2198 | 2570 | 2942 |
| 38 | 2351 | 2744 | 3138 |
| 39 | 2506 | 2922 | 3337 |
| 40 | 2665 | 3102 | 3539 |
| 41 | 2827 | 3285 | 3743 |
